# Combined Therapy Using Mesenchymal Stem Cells and Metal Nanoparticles: Perspectives for Ocular Injuries and Diseases

**DOI:** 10.2147/IJN.S527928

**Published:** 2025-06-12

**Authors:** Vladimir Holan, Eliska Javorkova, Barbora Hermankova, Pavel Rossner

**Affiliations:** 1 Department of Toxicology and Molecular Epidemiology, Institute of Experimental Medicine of the Czech Academy of Sciences, Prague, Czech Republic; 2 Department of Cell Biology, Faculty of Science, Charles University, Prague, Czech Republic

**Keywords:** mesenchymal stem cells, metal nanoparticles, combined application, ocular disorders, therapeutic effect

## Abstract

Stem cell-based therapy represents a promising approach for the treatment of numerous currently uncurable diseases. However, wider application of this therapy is still bound by various limitations. To increase the effectiveness of cell therapy, a combined application of stem cells with various types of chemicals or agents, which could support the immunoregulatory and therapeutic properties of stem cells, has been proposed and tested. One prospective approach is offered by the co-application of mesenchymal stem cells (MSCs), which have potent immunomodulatory and regenerative properties, and selected metal nanoparticles (NPs) which have been used in various fields of medicine for their immunomodulatory, anti-oxidant and antibacterial properties. It has been shown that the main mechanism of the therapeutic action of MSCs is the production of immunomodulatory molecules and growth factors, and that the secretory activity of MSCs can be modified by different types of NPs. For this purpose, metal NPs are extremely useful. They possess unique characteristics and can influence the growth and repair of tissues, exert strong antimicrobial activity and serve as nanocarriers. Thus, treatment based on the simultaneous application of MSCs and selected NPs combines the therapeutic effects of MSCs and impacts of NPs on applied MSCs, and on the cells and tissues of the recipient. In this review we outline the current state of studies combining the administration of MSCs and the application of metal NPs, with a focus on perspectives to use such treatment for corneal and retinal injuries and diseases.

## Graphical Abstract

**Figure:**
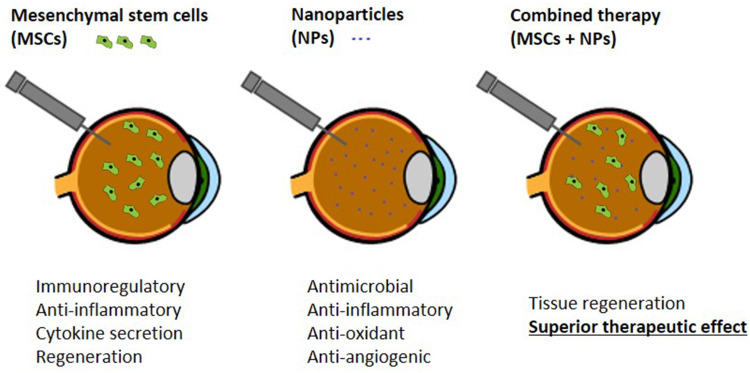

## Introduction

Ocular injuries and diseases represent the main cause of decreased quality of vision and even blindness world-wide. Despite the extensive experimental research and numerous preclinical and clinical trials, there is still an absence of efficient treatment protocols for the majority of these conditions. Although ocular diseases are a highly heterogenous group of disorders, a progressive decrease in the number or even the absence of specialized corneal or retinal cells is a common cause of these sight-threatening conditions. The important factors contributing to the development and progression of ocular diseases are a local inflammatory reaction, formation of reactive oxide species (ROS) and increased production of various cytokines, which damage the structure and function of ocular tissues.^1–3^ From the reason of heterogeneity and the often nonrecognized primary cause of ocular diseases, there is still an absence of effective therapeutic protocols. For the treatment of the damaged ocular surface, transplantation of corneal graft is the most common procedure.^4^ The main limiting factors for this therapy are a shortage of the grafts and the occurrence of limbal stem cell deficiency (LSCD) in cases of severe damage, which represents an obstacle for successful corneal regeneration.^5^ Similarly, treatment options for retinal degenerative diseases are still very limited. In the advanced stages, laser photocoagulation, vitrectomy or different microsurgery interventions, which represent complicated and highly invasive procedures. Less invasive treatments based on the administration of inhibitors of vascular endothelial growth factor (VEGF) or different low molecular weight drugs have been tested.^6–8^ However, these techniques and inhibitors have short-term effects and only slow down progression of the disease.

The most modern and prospective therapeutic approaches are based on the gene therapy or the application of stem cells.^9^,^10^ Among the different types of stem cells tested to date, the optimal properties were found in mesenchymal stem cells (MSCs). These cells possess potent immunoregulatory properties and can inhibit a harmful immune reaction at the inflammatory site of the injury or disease.^11^,^12^ In addition, MSCs produce numerous growth and neuroprotective factors which can support the survival of ocular cells and regeneration of diseased tissue. However, MSC therapy also has limitations and therefore new, and more effective and prospective approaches are being proposed and tested. One such option is represented by the application of MSCs in combination with selected agents which have proper immunoregulatory and therapeutic properties. Such support for MSC-based therapy can be provided by selected metal nanoparticles (NPs) which have potent antimicrobial, anti-oxidative, anti-angiogenic, and anti-inflammatory properties.^13^ Due to their small size, NPs can penetrate the cell membrane and influence cell functions. Following the simultaneous application of MSCs and NPs, NPs can also affect the properties of MSCs and directly influence the cells and tissues of the recipient. Thus, NPs could compensate for some of the deficiencies in stem cell therapy, and an appropriate combination of MSCs and selected NPs could be more effective than a single monotherapy with MSCs or NPs.

### Mesenchymal Stem Cells

MSCs represent a population of adult stem cells, which are present in nearly all tissues of the body. They are most frequently isolated from bone marrow or adipose tissue, and due to their good growth properties, are relatively easy propagated in vitro to obtain a sufficient number of cells. The cells are characterized according to their adherence to plastic surfaces, on the basis of phenotype markers, and by their ability to differentiate along the mesenchymal cell line.^14^ MSCs are potent producers of numerous immunoregulatory molecules and growth factors. Some of these factors, such as interleukin (IL)-6, transforming growth factor-β (TGF-β), hepatocyte growth factor (HGF) or VEGF, are produced spontaneously and their production can be increased after stimulation with mitogens or cytokines.^15^ On the contrary, the production of some other factors is rather decreased in the presence of proinflammatory cytokines.^15^ Using an appropriate combination of factors, the production of cytokines by MSCs can be intentionally enhanced or decreased.^16^,^17^ The regulation of the production of cytokines and growth factors by MSCs is important for their regulatory effects on cells of the immune system and for regenerative properties. As an example, according to the concentrations of cytokines in the environment, MSCs produce an enhanced or decreased amount of IL-6 or TGF-β. A mutual ratio of concentrations of these two cytokines regulates the development of proinflammatory T cells (higher levels of IL-6) or inhibitory regulatory T cells (higher concentrations of TGF-β).^18^,^19^ Another paracrine mechanism of MSC action is represented by the release of extracellular vesicles (exosomes) which share numerous function properties with MSCs.^20^,^21^ In addition to the secretory activity, MSCs contribute to immunoregulation and tissue regeneration by the expression of various intracellular or cell-bound regulatory molecules (such as indoleamine 2,3-dioxygenase, cyclooxygenase-2, Fas-L), by their anti-apoptotic properties, and by their ability to differentiate into other cell types.^11^,^22^

Using multiple mechanisms, MSCs inhibit T and B cell proliferation, production of cytokines by other cell types, suppress cytotoxic activity of T and NK cells, support development of regulatory T cells and attenuate inflammatory reactions.^11^,^23^ It has been shown in studies in vivo that administration of MSCs prolongs the survival of skin and organ allografts, decreases the incidence of graft-versus-host reaction, supports wound healing, attenuates septic complications, and suppresses the incidence and manifestation of various autoimmune diseases.^24–27^ In general, the effects of MSCs are mainly immunosuppressive and thus they can be used to inhibit undesirable immune reactions, including an inflammatory reaction in the diseased eye. On the other hand, numerous growth and trophic factors produced by MSCs can stimulate tissue growth and support survival and regeneration of the diseased tissue. Therefore, these properties make MSCs suitable candidates for the treatment of so far uncurable ocular diseases. This conclusion is supported by observations from both experimental and clinical studies, that administration of MSCs is safe and does not induce tumour growth during long-term observation, and is without significant negative side effects.^28^,^29^

Despite the numerous advantages of MSCs for therapeutic use, stem cell therapy is hampered by several issues, such as poor MSC survival after cell transplantation,^30^,^31^ insufficient homing to the injured site after systemic administration, the dependence of the secretory activity of transplanted MSCs on the cytokine environment in the diseased tissue,^16^ and the different and often unknown mechanisms of MSC action.^32^,^33^

### The Use of MSCs for the Treatment of Ocular Injuries and Diseases

Injuries or diseases of the cornea represent one of the main causes of decreased quality of vision or even blindness. Classical therapy of the damaged ocular surface is based on transplantation of the corneal allograft, which represents a relatively successful procedure. However, if the damage of the ocular surface is more extensive and the limbal region is involved, the state of LSCD is induced and the cornea cannot be naturally regenerated. In such cases, the only method for the treatment of LSCD is transplantation of the limbal stem cells (LSCs).^34^,^35^ Since the isolation and cultivation of autologous LSCs has numerous limitations, such as the absence or a low number of autologous cells, allogeneic LSCs or other sources of stem cells have been tested. Among them, MSCs for their anti-inflammatory and regenerative properties, have turned out to be the most promising cell population. To date, various types of MSCs and different methods of their application have been tested. In a rabbit model of chemically damaged ocular surface, the regenerative properties of bone marrow-derived MSCs, adipose tissue-derived MSCs and LSCs were compared.^36^ In this study, MSCs had a comparable therapeutic effect, as had LSCs. However, MSCs cannot be applied onto the ocular surface directly, but must be fixed using various scaffolds such as nanofibers, amniotic membranes, contact lenses or fibrin glue.^37^ In the first experiments, MSCs speeded up regeneration and prevented a local inflammatory reaction in the models of chemically or mechanically damaged ocular surface and LSCD.^38–40^ The experiments with transplantation of MSCs yielded promising results and thus the first clinical trials were reported. The safety of the procedure and the improvement in the corneal surface structure and in visual acuity have been described in MSC-treated patients suffering from LSCD, ocular burn, dry eye or severe keratoconus.^41–44^

Another group of serious ocular disorders is represented by retinal degenerative diseases. This group includes sight-threatening diseases, such as age-related macular degeneration (AMD), retinitis pigmentosa (RP), diabetic retinopathy (DR), glaucoma and some other, rarely occurring diseases. Although these diseases have various primary causes and different aetiologies, a common characteristic is the dying of specialized retinal cells and a local inflammatory reaction. Therefore, inhibition of the inflammation and the support for the surviving retinal cells appear to be prospective approaches for the treatment. To date, various stem cell types with immunoregulatory and anti-inflammatory properties and with the potential to produce growth factors have been proven as a promising tool to treat these diseases.^10^,^45^ It has been shown in different models of retinal diseases that intraocular administration of MSCs alleviated the local inflammatory reaction, slowed down the progression of the disease and improved the architecture and function properties of the diseased retina.^46–48^ The promising results from experimental studies stimulated clinical trials in patients suffering from retinal diseases. The first conclusions in patients with RP, DR or AMD showed the safety of the treatment and improvement of visual acuity.^49^,^50^ MSCs can be therapeutically applied by different modes. In addition to systemic administration through intravenous route, for the treatment of ocular surface disorders, a topical application using different carriers to fix the cells or application into the lacrimal gland, are the most common methods. In cases of retinal diseases, intravitreal, retrobulbar or subretinal implantations have been used. However, the optimal method of MSC application depends on the type and state of the disease.

MSC therapy has been proven in experimental studies and clinical trials to also be beneficial for the treatment of other ocular diseases, such as symptoms of dry eye, keratoconus, viral or bacterial infections, autoimmune diseases and others.^44^,^51^,^52^ Selected studies showing the therapeutic effect of MSCs on different ocular disorders in experimental models and clinical trials are summarized in Table 1.

**Table 1:** Selected Experimental and Clinical Studies on the Therapeutic Effects of MSCs for Ocular Injuries and Diseases

Recipient	Disease	MSCs	Application	Result	Reference
Mouse	DED	BM-MSCs	Subconjunctival	Alleviation of the disease	[53]
Mouse	Alkali burned cornea	BM-MSCs	Topical	Suppression of inflammation	[54]
Rabbit	Alkali burned cornea	BM-MSCs	Topical	Improvement of healing of corneal architecture	[36]
Rat	Retinal degeneration	BM-MSCs	Epiretinal	Restored retinal and visual functions	[55]
Rat	Glaucoma	BM-MSCs	Intravitreal	Preservation of retinal ganglion cell function	[56]
Human	Keratoconus	AT-MSCs	Intrastromal	Improvement of visual acuity	[44]
Human	AMD	BM-MSCs	Retrobulbar	Improvement of visual acuity	[50]
Human	Sjögren´s syndrome and DED	AT-MSCs	Lacrimal gland	Improvement of DED	[57]

**Abbreviations**: BM-MSCs, bone marrow-derived MSCs; AT-MSCs, adipose tissue-derived MSCs; AMD, age-related macular degeneration; DED, dry eye disease.

Although stem cell-based therapies have turned out to be a promising therapeutic approach, there are still possibilities to potentiate the beneficial effects of this treatment. One such prospective approach is based on the co-application of therapeutic MSCs and metal NPs, which have antimicrobial and anti-inflammatory properties and influence cell functions.

### Metal Nanoparticles

NPs represent very small elements (1–100 nm in size) which can be derived from both natural and synthetic sources and can be prepared to the required size and shape.^58^ Due to their small size NPs can penetrate even very small capillaries through the body and readily enter the cell where they induce numerous molecular changes. This is especially important in ophthalmology, where classical therapy is restricted by various anatomical and physiological ocular barriers, which prevent the penetration of cells or drugs into the diseased site.

Among the various types of NPs, prepared from different materials, the most studied group is represented by metal NPs. They are made of pure metals, such as silver (Ag), gold (Au), zinc (Zn), titanium (Ti), copper (Cu), platinum (Pt), cerium (Ce), or iron (Fe), or their compounds like oxides, sulphides, chlorides, and phosphates. Due to their unique properties, including amenable functionalization and stability, they can be modified with different targeting agents and thus they hold great promise in biomedical research and applications.^59^ Metal NPs have antibacterial properties and thus they have been proposed as an alternative over traditional antibiotics to overcome bacterial resistance.^60^,^61^ In ophthalmology, bacteria are the major contributors of ocular infections worldwide.^62^ If left untreated, bacterial infections can damage the structures of the eye tissue with possible visual impairments. With increasing incidence of bacterial resistance to antibiotics metal NPs have perspective in ocular therapy. In addition, some metal NPs can combat oxidative stress or scavenge ROS, or are commonly exploited for their antifungal, antiviral, anti-angiogenic or anti-inflammatory effects.^13^,^63^ Despite great promise and advantages, the clinical application of various NPs, nanocarriers and nano contrast agents, is still met with several challenges, such as potential cytotoxicity, batch-to-batch variations, and often nonbiodegradability.

Irrespective of the persisting shortages and disadvantages of some NP products, it has been shown in experimental studies that application of NPs in vivo has an impact on various physiological functions,^64^,^65^ including the reactivity of cells of the immune system^66^,^67^ or on healing processes in the eye.^13^,^68^,^69^ Therefore, therapeutic application of NPs has to be carefully considered and used with a precaution.

### The Impacts of Metal NPs on Stem Cells

The outcome of combined treatment based on the simultaneous application of therapeutic MSCs and metal NPs, especially in the case of local application, can be influenced by the effect of NPs on MSCs. Numerous studies have demonstrated both the negative and positive influence of NPs on the viability, differentiation potential and function activity of different types of stem cells, including embryonic stem cells (ESCs) and various types of adult stem cells.

Studies using mouse and human ESCs showed a dose-dependent toxic effect, which was influenced by the type and size of NPs. For example, Gao et al^70^ showed that Ag NPs affected the expression of a large number of genes in mouse ESCs. The authors concluded that these transcriptomic changes could exert an adverse effect on the functions of ESCs. Similarly, toxic effects of Ag NPs on mouse ESCs were described by Rajanahalli et al^71^ who concluded that the toxic effects of these NPs on stem cells were due to the overproduction of ROS, which altered the gene expression and protein modifications. Another study showed the toxic effects of Au NPs on human ESCs and the toxicity depended on the NP size and surface chemistry.^72^ On the contrary, a study by Wei et al^73^ showed that Au NPs rather enhanced differentiation of mouse ESCs into dopaminergic neurons.

Toxic effects of metal NPs were also reported in studies using adult stem cells, such as human hematopoietic stem cells^74^ and mouse spermatogonial stem cells.^75^ The first studies on the effects of metal NPs on MSCs showed toxic effects of Ag NPs on the growth and differentiation of human MSCs^76^ or ZnO NPs on rat adipose tissue-derived MSCs.^77^ On the contrary to the negative effects of NPs on stem cells described above, other authors demonstrated rather positive impacts of some metal NPs on MSCs. For example, Zhang et al^78^ observed that Ag NPs promoted MSC proliferation and differentiation in a mouse model. Similarly, a stimulatory effect of Ag NPs on the osteogenesis of human MSCs was described by He et al^79^ and Yi et al^80^ reported similar promoting effects of Au NPs. The studies in vitro suggested that the effects of NPs depend on a number of factors, including original material used for preparation of NPs, particle size, shape, surface charge, surface coating, solubility, concentration, growth media and exposure time.^81^,^82^ Among the different types of NPs prepared from distinct starting materials, the most toxic NPs are those made from metals, as was documented in different models.^83^,^84^ It was shown that CuO NPs, Ag NPs and ZnO NPs belong among the most toxic particles, while TiO_2_ NPs are the least toxic.^85–87^ In a direct comparison of four different types of metal NPs (CuO NPs, Ag NPs, ZnO NPs, and TiO_2_ NPs) in a model of mouse MSCs as target cells, we demonstrated that CuO NPs were the most toxic and TiO_2_ NPs were the least cytotoxic.^88^ Similarly, apparent differences in the effects of individual metal NPs were obtained when we tested the impacts of NPs on the immunoregulatory and therapeutic properties of MSCs.^89^ Although Ag NPs and CuO NPs were the most toxic in higher concentrations, these NPs rather enhanced some MSC functions in low NP concentrations.^89^ Similar observations were reported by Algazlan et al^90^ who tested the effects of Ag NPs on the properties of human MSCs. Thus, published results on the toxic effects of individual NPs on stem cells have so far been contradictory. Therefore, optimal types of NPs have to be carefully selected and tested before their therapeutic use in vivo.

### The Use of Metal NPs in Ophthalmology

Metal NPs have gained a lot of attention in medicine for their potent antibacterial, anti-angiogenetic and anti-inflammatory properties. In ophthalmology, these particles also deserve attention for their potential to cross the barriers in the eye, and for the ability to serve as antibacterial agents with a perspective to replace classical antibiotics in the cases of the need to overcome bacterial resistance.

One group of metal NPs is represented by materials with antimicrobial properties. Typical representatives are Ag NPs and Au NPs. Au NPs also have exceptional chemical stability and good bioconjugation properties making them suitable carriers for various therapeutics and for drug delivery. For example, the incorporation of N-acetylcarnosine into Au NPs improved the therapeutic effects of this drug in cataract treatment.^91^ Ag NPs are known in biomedicine mainly for their antimicrobial properties and their ability to inhibit angiogenesis. In an animal experimental model of selenium cataractogenesis, it was demonstrated that Ag NPs exhibit antioxidant activity and have potent anticataract effects.^92^ In a mouse model of cytokine-induced inflammatory reaction in the retina, the intravitreal administration of Ag NPs significantly reduced the activation of microglia and inhibited a local inflammatory reaction, but did not influence the expression of the gene for rhodopsin by retinal cells.^93^ Zhang et al^94^ showed that CuO NPs mitigated retinal vasculature development and alleviated pathological retinal angiogenesis in vitro and in vivo. The authors concluded that CuO NPs may be an effective anti-angiogenic agent for the treatment of retinal angiogenesis.

On the contrary, TiO_2_ NPs administered into the eye did not induce any toxicity at the level of gene expression and histological integrity of the retina and did not affect the viability of retinal cells. However, these NPs administered intravitreally suppressed retinal neovascularization in oxygen-induced retinopathy in mice.^95^

Cerium oxide (CeO_2_) NPs have gained considerable attention in the field of biomedicine as anti-oxidant material with potential medical applications. Cerium is known as a scavenger of ROS, and thus it is an appealing biomaterial for protecting against cataract formation.^96^ In this respect, Hanafy et al^97^ described that CeO_2_ NPs, by their multiple mechanisms, inhibit cataract progression in human lens, and Yang et al^98^ showed that administration of cerium chloride-loaded mesoporous silica NPs significantly antagonized oxidative stress in streptozotocin-induced diabetic cataract rats, thus alleviating the development and progression of cataracts. Similarly, cerium oxide nanocrystals in combination with MSC exosomes scavenge ROS, suppress inflammation and alleviate dry eye symptoms.^99^ In addition, intravitreally applied CeO_2_ NPs reduce microglial activation and neurodegeneration processes in light-damaged retina in a rat model.^100^

Selected studies on the therapeutic effect of metal NPs for ocular diseases and disorders are shown in Table 2.

**Table 2:** Selected Studies Using Application of Metal NPs for the Treatment of Ocular Disorders

Recipient	Disease	NPs	Application	Result	Reference
Mouse	Oxygen-induced retinopathy	TiO_2_	Intravitreal	Suppression of neovascularization	[95]
Mouse	DED model	CeO_2_	Superficial	Improvement of DED symptoms	[101]
Mouse	Light-induced retinal degeneration	Pt	Intravitreal	Reduction of ROS, protection of the retina	[102]
Mouse	Cytokine-induced retinal inflammation	Ag	Intravitreal	Inhibition of microglia activation, alleviation of inflammation	[93]
Rat	Light-damaged retina	CeO_2_	Intravitreal	Suppression of retinal neurodegeneration	[100]
Mouse	Oxygen-induced retinopathy	CuO	Intravitreal	Alleviation of retinal angiogenesis	[94]

**Abbreviations**: NPs, nanoparticles; DED, dry eye disease; ROS, reactive oxygen species.

### Combined Application of MSCs and NPs

The promising therapeutic effects of a single therapy based on the application of MSCs or NPs has stimulated interest in combined therapy which could provide additive or synergistic effects, and could be a superior approach in comparison with a single therapy. The data published to date on the treatment of eye diseases using combined application of MSCs and NPs are nearly absent or very limited, but the results from other experimental models have shown advantages of this type of treatment.

One type of such approach is based on the application of MSCs loaded with NPs or the use of MSC-derived exosomes and NPs. In an eye model, Tian et al^99^ showed that CeO_3_ nanocrystals prepared on MSC exosomes were more effective than a single therapy with CeO_3_NPs or MSC exosomes alone, in the suppression of ROS formation and inflammation in a model of chemically induced dry eye disease in mice. Other models pointed out that MSCs loaded with NPs could serve as an effective delivery system and are more effective than MSCs or NPs alone. For example, Cheng et al^103^ showed in a rat experimental model that the application of MSCs loaded with Au NPs could represent a biosafe nanodrug delivery system. Recent advances in the combination of stem cell therapy and various NPs after spinal cord injuries were summarized and discussed by Garcia et al.^104^ The authors concluded that the selection of an optimal type of NPs and stem cells is crucial before the use of this approach in clinical trials.

Promising results of combined therapy were also obtained in models where MSCs and NPs were administered separately. In a rat tibial fracture model, Chen et al^105^ showed that the fracture healing was significantly accelerated in the group treated simultaneously with MSCs and Pt NPs in comparison with the control group or groups treated with MSCs or Pt NPs alone. In a model of ischemic stroke in rats, Nazarian et al^106^ showed that combined treatment with Au NPs and MSCs administered at the same time had a better therapeutic effect on ischemic brain injuries than a single therapy. In another model, the transplantation of MSCs combined with application of non-metallic selenium (Se) NPs or galantamine NPs was more effective in the neuroprotection in an Alzheimer´s disease model in rats than a conventional treatment with NPs or MSCs alone.^107^,^108^ So far, combined therapies with NPs and stem cells were successfully used for the treatment in experimental models of spinal cord injuries,^109^,^110^ for neuroprotection of streptozotocin-induced neurotoxicity in rats,^111^ inhibition of tumour growth^112^,^113^ or in experimental model of acute cerebral infarction.^114^ The current state and perspectives of the research on nanomaterials combined with MSCs for the treatment of ischemic stroke and neurodegenerative diseases were recently reviewed and discussed by Wei et al^63^ and Xu et al.^115^ The authors concluded that although various obstacles and challenges still remain, therapies based on the simultaneous application of stem cells and NPs represent a promising approach for the treatment of currently incurable diseases.

NPs can be used for their antibacterial and anti-inflammatory properties, but could also serve as carriers of different therapeutic agents. In addition to their therapeutic effects, NPs could act as targeting agent to deliver stem cells to injured tissue. For example, MSCs labelled with superparamagnetic iron oxide NPs can be targeted using external magnetic field.^110^,^116^ The combined application of MSCs and NPs can not only benefit from the therapeutic features of MSC or NP single therapies, but can also improve limitations of single therapy. This conclusion is supported by the to date published studies from different experimental models demonstrating superior therapeutic effects of combined therapy in comparison with a monotherapy using MSCs or NPs alone. Selected studies based on the separate application of MSCs and NPs are shown in Table 3.

**Table 3:** Selected Studies Based on the Separate Application of MSCs and NPs for the Treatment of Serious Disorders

Recipient	Disease	MSCs + NPs	Application	Result	Reference
Mouse	DED	BM-MSCs + CeO_3_ NPs	Topical + topical	Alleviation of DED	[99]
Rat	Tibial fracture	AT-MSCs + Pt NPs	Local + oral	Accelerated healing	[105]
Rat	Ischemic stroke	AT-MSCs + Au NPs	Local + oral	Positive effect on brain injury	[106]
Rat	Alzheimer´s disease model	AT-MSCs + Se NPs	ICV + oral	Neuroprotection	[107]

**Abbreviations**: MSCs, mesenchymal stem cells; BM-MSCs, bone marrow-derived MSCs; NPs, nanoparticles; DED, dry eye disease; AT-MSCs, adipose tissue-derived MSCs; ICV, intracerebroventricular.

## Conclusions and Perspectives

The published data have shown the potential and current perspectives of combined therapy with MSCs and metal NPs for the treatment of different types of disorders, including ocular diseases. Although single therapies with MSCs or NPs have clinical potential, in the case of MSC therapy there are limitations associated with the standardization of MSC preparation, in the relative short life-span of MSCs, dependence of secretory activity on the environment and restricted penetration of cells through ocular barriers. On the contrary, NPs due to their small size can easily pass through anatomic barriers and penetrate cells and have potent antibacterial and antiviral properties. For their antimicrobial activity, NPs have huge biomedical potential in situations when microbes resistant to classic antibiotics are involved in the disease.^61^ However, the main disadvantage of therapy with metal NPs is their toxicity, if used in higher concentrations. Since the properties of NPs strongly depend on their structure, the preparation of less toxic, but still effective NPs will be a big task for their future production. The combination of MSCs and NPs could decrease concentrations of NPs needed for the effective therapy or to compensate for some of the deficiencies associated with a single therapy. Another advantage of the combined therapy for ocular surface diseases consists in the possibility to administer MSCs (using various carriers) topically on the ocular surface,^117^,^118^ where MSCs inhibit a local inflammatory reaction and support healing process. Similarly, NPs can be applied directly on the ocular surface,^119^,^120^ where they penetrate into ocular tissue and mediate antimicrobial effects. Thus, simultaneous application of MSCs and NPs can have synergistic/additive therapeutic potential.

The beneficial effects of a single therapy with MSCs or NPs for ocular disorders have been well documented in numerous models.^13^,^36^,^45^,^97^ However, published data showed that single therapies with MSCs or NPs have their advantages and disadvantages, and the combined treatment based on simultaneous administration of MSCs and NPs has turned out to be superior over a single therapy. Although currently there is a limited number of publications on combined therapy with MSCs and NPs, the first studies in ophthalmology^99^ and in other models^105–107^ have confirmed the promise and perspectives of this approach. Nevertheless, in a simultaneous local application of MSCs and NPs there could be a danger of negative effects of NPs on therapeutically administered MSCs^89^,^121^ and thus the types and doses of NPs or MSCs have to be carefully considered and tested before clinical use.

## Abbreviations

AT-MSCs, adipose tissue-derived MSCs; AMD, age-related macular degeneration; BM-MSCs, bone marrow-derived MSCs; DED, dry eye disease; ESCs, embryonic stem cells; HGF, hepatocyte growth factor; ICV, intracerebroventricular; IL, interleukin; LSCD, limbal stem cell deficiency; LSCs, limbal stem cells; MSCs, mesenchymal stem cells; NPs, nanoparticles; ROS, reactive oxygen species; RP, retinitis pigmentosa; TGF, transforming growth factor; VEGF, vascular endothelial growth factor.
